# Nitric oxide scavenging by red cell microparticles and cell free hemoglobin as a mechanism for hemolytic diseases and the red blood cell storage lesion

**DOI:** 10.1186/1471-2210-11-S1-O7

**Published:** 2011-08-01

**Authors:** Marc T Gladwin

**Affiliations:** 1 University of Pittsburgh, Vascular Medicine Institute, Pittsburgh, USA; 2 University of Pittsburgh, Allergy and Critical Care Medicine, School of Medicine, Pittsburgh, USA

## 

Intravascular red cell hemolysis impairs NO-redox homeostasis, producing endothelial dysfunction, platelet activation and vasculopathy. A central mechanism is that cell-free plasma hemoglobin reacts with the vasodilator nitric oxide (NO) in a 1:1 stoichiometric reaction that occurs at high rate of 6-8x107 mol/L/s. This consumption of NO by cell free plasma hemoglobin has been shown to cause changes in vascular function in subjects with sickle cell anaemia who had plasma heme concentrations as low as 6 µM. Approximately half of the observed “hemolysis” in stored blood is also encapsulated in microparticles, and, in theory, based on their small molecular size, microparticles may also exhibit accelerated NO scavenging rates compared with intact erythrocytes. The effect of microparticles on NO scavenging could be especially important, particularly in the microvasculature where their clearance may not be reflected by systemic clearance rates. Red blood cell storage under standard blood banking conditions results in reduced integrity of the erythrocyte membrane, with formation of exocytic microvesicles or “microparticles” and hemolysis, which we hypothesized could impair vascular function and contribute to the putative “storage lesion” of banked blood. We now find that storage of human red blood cells under standard blood banking conditions results in the accumulation of cell free and microparticle-encapsulated hemoglobin which, despite 39 days of storage, remains in the reduced ferrous oxyhemoglobin redox state and stoichiometrically reacts with and scavenges the vasodilator NO. Using stopped-flow spectroscopy and laser triggered NO release from a caged NO compound we found that both free hemoglobin and microparticles react with NO about 1000 times faster than with intact erythrocytes. In complementary in vivo studies we show that hemoglobin, even at concentrations below 10 µM (in heme), produces potent vasoconstriction when infused into the rat circulation, while controlled infusions of methemoglobin and cyanomethemoglobin, which do not consume NO, have substantially reduced vasoconstrictor effects. Infusion of the plasma from stored human red cell units into the rat circulation produces significant vasoconstriction related to the magnitude of storage related hemolysis. Finally, we explored new therapies that have the potential to bypass NO scavenging via direct pharmacological activation of soluble guanylyl cyclase (sGC). These sGC activators or stimulators show promise in the reversal of free-hemoglobin mediated vasoconstriction. The results of these studies suggest new mechanisms for endothelial injury and impaired vascular function associated with the most fundamental of red blood cell storage lesions, hemolysis, as well as suggest a new approach to therapeutic restoration of cGMP levels in the setting of NO scavenging reactions.

